# Next‐Generation Sequencing in Infectious‐Disease Diagnostics: Economic, Regulatory, and Clinical Pathways to Adoption

**DOI:** 10.1002/mbo3.70104

**Published:** 2025-11-26

**Authors:** John Osei Sekyere

**Affiliations:** ^1^ Department of Medical Microbiology, School of Medicine University of Pretoria Pretoria South Africa; ^2^ Institute of Biomarker Research and Department of Clinical Development Medical Diagnostic Laboratories LLC Hamilton Township New Jersey USA

**Keywords:** antimicrobial resistance, clinical genomics, health economics, infectious disease diagnostics, insurance reimbursement, next‐generation sequencing, polymerase chain reaction, syndromic testing, targeted amplicon sequencing, whole‐genome sequencing

## Abstract

Next‐generation sequencing (NGS) has emerged as a transformative tool for infectious disease diagnosis, offering broad pathogen detection, antimicrobial resistance profiling, and syndromic panel testing. However, widespread clinical adoption remains hindered by insurance reimbursement challenges, high costs, and regulatory barriers. Unlike polymerase chain reaction (PCR), which enjoys well‐established Current Procedural Terminology (CPT) codes and reimbursement pathways, many NGS‐based tests lack standardized billing mechanisms, discouraging laboratories from integrating NGS into routine diagnostics. This article explores the economic, clinical, and technological considerations of targeted amplicon sequencing (tNGS) versus PCR and whole‐genome sequencing (WGS), demonstrating how optimized multiplexing strategies, emerging NGS platforms, and regulatory advancements can enhance feasibility. It is argued that insurance policies must evolve to recognize NGS's superior clinical utility in detecting polymicrobial infections, emerging pathogens, and antimicrobial resistance determinants, ultimately improving patient outcomes and reducing healthcare costs. Current reagent‐only costs now average US $65 per microbial genome, US $600 per 30× human genome, and US $130–600 per metagenomic sample when multiplexed; these figures continue to fall with higher multiplexing. To accelerate equitable adoption, we recommend near‐term payer coverage pilots for clearly defined clinical indications, dedicated CPT pathways for infectious‐disease sequencing (including metagenomic assays), and pragmatic validation frameworks that acknowledge genotype–phenotype limits while leveraging multiplexing and centralized reference workflows.

## Graphical abstract

Next‐generation sequencing (NGS) is poised to transform infectious‐disease diagnostics. Falling per‐sample costs, new CPT codes and FDA pathways, and 24‐h, multiplexed resistome reports converge to deliver faster, evidence‐based therapy and stronger surveillance. Our roadmap links the economic, regulatory, and clinical pillars to accelerate routine NGS adoption and improve patient outcomes.

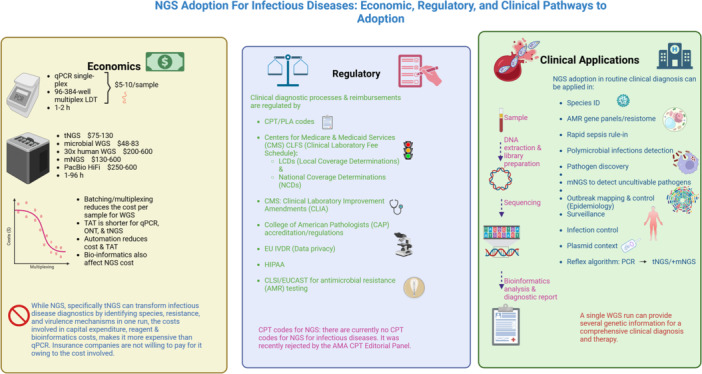

## 1 Introduction

The increasing burden of infectious diseases and the rapid evolution of antimicrobial resistance (AMR) necessitate more comprehensive and efficient diagnostic approaches (Antimicrobial Resistance Collaborators 2022). PCR remains the gold standard for pathogen detection, but its inability to assess multiple pathogens efficiently in syndromic infections limits its utility (Patel 2016). NGS, particularly targeted amplicon sequencing (tNGS), provides a high‐throughput alternative capable of identifying multiple pathogens and their resistance genes in a single assay (Chiu and Miller 2019).

NGS chemistries differ from platform to platform. In short‐read platforms, fragmented DNA libraries are clonally amplified and imaged over iterative synthesis cycles to yield millions to billions of short reads that are aligned to references or assembled *de novo*. Long‐read platforms sequence single molecules in real time, either by measuring ionic current changes across nanopores (Oxford Nanopore) or by monitoring fluorescently labeled nucleotides incorporated by a polymerase in zero‐mode waveguides (PacBio HiFi). Long reads simplify genome closure, structural‐variant and plasmid detection, and resistance gene context, whereas short reads offer high throughput and low per‐base cost at scale.

Despite its potential, NGS adoption in clinical microbiology remains slow due to three primary barriers: high per‐sample costs, long turnaround times (TAT), and, most critically, the lack of insurance reimbursement codes (Schlaberg et al. 2017). Traditional diagnostic workflows often require sequential testing using pathogen‐specific PCR assays, leading to delayed diagnosis, increased healthcare costs, and potentially suboptimal treatment strategies (Schlaberg et al. 2017; Su 2025; He 2023). The COVID‐19 pandemic has highlighted the value of comprehensive molecular diagnostics and the limitations of single‐target approaches, presenting an opportunity to reconsider current diagnostic paradigms (Zhu et al. 2020).

In 2019, an estimated 1.27 million deaths were directly attributable to antibiotic‐resistant bacterial infections, and a further 4.95 million were associated with AMR, underscoring the urgency for innovative diagnostics (Antimicrobial Resistance Collaborators 2022). Thus, next‐generation sequencing (NGS) technologies have never been more critical for early, accurate pathogen detection and effective antimicrobial stewardship.

This article examines the barriers to NGS implementation in clinical infectious disease diagnostics and proposes strategies to integrate NGS into routine workflows while maintaining cost‐effectiveness. It is emphasized that addressing reimbursement challenges is crucial to realizing the full potential of NGS in improving patient care and antimicrobial stewardship. Throughout this review, we focus on the United States reimbursement landscape between January 2023 and March 2025, unless otherwise stated, because insurance coding, pricing, and regulatory pathways differ substantially across jurisdictions. International experience is summarized later to provide contrast and transferable lessons.

### 1.1 Evidence Before This Article

A comprehensive search on PubMed for English‐language articles (up to 2025) on next‐generation sequencing (NGS) in infectious disease diagnosis, focusing on systematic reviews, key clinical studies, and policy analyses was conducted. The existing evidence indicates that untargeted metagenomic NGS can identify pathogens in cases where standard tests (culture or PCR) fail, thereby improving diagnostic yield (Wilson et al. 2019; MacFadden et al. 2020). A multicenter study showed that mNGS of cerebrospinal fluid provided etiological diagnoses in patients with unknown meningitis/encephalitis, prompting targeted therapies (Wilson et al. 2019). Additionally, targeted amplicon sequencing has successfully detected drug‐resistance mutations and mixed infections that might be missed by single‐pathogen assays (MacFadden et al. 2020).

Several systematic reviews and expert commentaries have highlighted both the potential and challenges of integrating NGS into clinical practice. Chiu and Miller (2019) reviewed the clinical applications of metagenomic NGS and noted its significant diagnostic advantages along with the need for streamlined workflows (Chiu and Miller 2019). A recent systematic review by Balloux et al. examined international experiences and found that while the clinical utility of NGS in infectious diseases is increasingly evident (Balloux et al. 2018), the lack of reimbursement and standardization remains a global barrier to routine use. This review and others emphasize that robust evidence of cost‐effectiveness and outcome improvement is crucial for broader adoption of NGS (Chiu and Miller 2019). Meanwhile, several national initiatives have demonstrated feasible models for implementation: for instance, South Korea's program for AMR surveillance via NGS (Kim et al. 2022) and Japan's nationwide clinical sequencing network (Tohya et al. 2022) both illustrate how targeted support and public‐private partnerships can overcome logistical and financial hurdles (Tohya et al. 2022; Kim et al. 2022). These initiatives report improved detection of outbreaks and resistance patterns, informing public health interventions where traditional methods were inadequate.

In summary, prior evidence establishes that NGS can substantially broaden diagnostic scope and improve patient outcomes in infectious diseases. However, at the time of this review, routine clinical adoption of NGS is limited. The literature prior to this article consistently pointed to high costs, slow turnaround, and particularly the absence of insurance reimbursement mechanisms as the major obstacles to implementation. Few, if any, Current Procedural Terminology (CPT) codes existed for comprehensive infectious disease sequencing, and payers often either denied coverage or limited payments to small PCR panels. Thus, before this analysis, there was a recognized need for strategies to demonstrate the value of NGS and to reform reimbursement policies, as well as real‐world examples (albeit scarce) of how such changes might be achieved in practice.

## 2 Economic Considerations: NGS vs PCR vs WGS

### 2.1 Comparative Cost Analysis

PCR remains the most widely used molecular diagnostic method due to its relatively low cost. In‐house PCR assays typically range from $5 to $10 per sample, making them a cost‐effective option for detecting single pathogens (Buchan and Ledeboer 2014). Commercial PCR kits provide standardization and ease of use at approximately $40 per test. Multiplex PCR panels, which detect multiple pathogens simultaneously, can cost between $100 and $200 per sample, depending on the number of targets included (Green et al. 2016) Large academic/tertiary laboratories frequently deploy 384‐well, miniaturized multiplex qPCR LDTs, enabling 384 samples per run with 1–3 h TAT and $3–8 reagent cost per sample when reactions are 5–10 µL, which materially lowers per‐test cost compared with cartridge panels.

In contrast, NGS costs vary based on the approach used. Whole‐genome sequencing (WGS) remains expensive, with reagent costs averaging around $600 per sample for a 30× human genome (Ellington et al. 2017) Targeted amplicon sequencing (tNGS), which focuses on specific genomic regions of interest, offers a more economical alternative, with reagent costs ranging between $75 and $130 per sample (Miao et al. 2018). Additional costs associated with library preparation and sequencing contribute to the overall expense, but multiplexing strategies can significantly reduce per‐sample costs.

Recent technological advances have dramatically reduced NGS costs, with some platforms enabling up to 96 samples to be processed simultaneously, potentially bringing per‐sample costs below $100 when fully optimized (Besser et al. 2018). This cost reduction makes tNGS increasingly competitive with multiplex PCR panels while offering broader pathogen coverage and resistance detection capabilities.

### 2.2 Additional Cost Factors Beyond Reagents

While reagent costs significantly influence per‐sample test pricing, labor, equipment depreciation, overhead, and data storage can also add several dollars per sample. A single sequencing run typically requires not only the reagents to prepare libraries but also the hands‐on technician time (often $6–$10 per sample), instrument amortization (e.g., $2–$5 per sample), and maintenance. Moreover, data storage and analysis must be factored in, particularly for facilities that keep raw NGS data for extended periods to comply with regulatory requirements.

### 2.3 NGSCost per Sample (2023–2025)

#### 2.3.1 Microbial Whole‐Genome Sequencing (Bacteria and Fungi)

The per‐sample cost of sequencing microbial genomes has dropped significantly. In research and public health laboratories, sequencing a bacterial genome can cost on the order of only tens of dollars in consumables when runs are highly multiplexed (Antimicrobial Resistance Collaborators 2022; Waggle et al. 2024). One recent hospital‐based WGS surveillance program reported reagent costs ranging from $48–$83 per bacterial isolate genome (averaging $65) when using an Illumina NextSeq, achieved by batching up to 96 samples per run (Waggle et al. 2024). Even after including labor and overhead, their all‐inclusive cost was under $100 per sample (Waggle et al. 2024). These lower costs reflect efficient multiplexing and workflow optimizations (e.g. using smaller reaction volumes) (Waggle et al. 2024). In contrast, clinical sequencing of a pathogen isolate in a diagnostic laboratory may be higher due to additional expenses for certified procedures and analysis; however, the continued improvements in technology are narrowing this gap. Overall, $50–$150 per genome is a reasonable current range for microbial WGS in bulk (lower end for large‐scale sequencing centers, higher end for clinical labs). Fungal genomes (often larger than bacteria) incur slightly higher sequencing costs due to needing greater read depth, but still generally fall well below the cost of human genome sequencing when multiplexed (Table 1).

**Table 1:** Typical batching, TAT, and per‐sample reagent costs for infectious‐disease sequencing workflows (U.S., 2024–2025). *Reagent cost* = lab consumables only; *Real‐world clinical cost* = typical U.S. test price billed/paid (where available). See footnotes and abbreviations.

Assay/Platform	Typical batching	Lab TAT	Throughput per run	Approx. reagent cost/sample (USD)*	Real‐world clinical cost†	Notes (platform, scale)	References‡
Single‐plex in‐house qPCR	96‐well	Same day	94–96	$5–10	$30–60	1–2 targets, 96‐well plate	CMS CLFS 2025; CLSI PCR
Commercial single‐plex PCR kit	Cartridge	1–2 h	1	~$40	$75–120	FDA‐cleared cartridges	Manufacturer IFUs; CMS CLFS 2025
Syndromic multiplex PCR panel (12–25 targets)	Cartridge	1–2 h	1	$100–200	$300–700	e.g., BioFire RP2.1	BioFire RP2.1 IFU; payer LCD
High‐throughput multiplex qPCR (LDT)	384‐well	Same day	368–384	$3–6	$20–50	Laboratory‐developed test (LDT); batch to capacity	CLSI MM17; CAP Molecular Checklist
Targeted amplicon NGS (tNGS)	48–96	1–2 d	48–96	$75–130	$200–400	MiSeq v2 class; barcoded pools	Waggle 2024; Eyre 2024
Microbial isolate WGS (short‐read)	96	1–2 d	96	$48–83	$90–150	96 isolates/NextSeq‐class	Waggle 2024; Eyre 2024
Human 30× WGS (short‐read)	384–1,000	1–2 d	384–1,000	~$200–600	$500–3,400	NovaSeq X class; technical‐only offerings from ~$499; interpreted diagnostic tests higher	Illumina 2023; Broad Clinical Labs 2025
Metagenomic NGS (mNGS)	8–24	1–3 d	8–24	$130–600	$1,000–2,500	Depth‐dependent; host depletion often required	Wilson/Chiu 2024; program pricing
ONT long‐read (MinION/PromethION)	1–96	Same day	1–96	$100–400 (per barcode)	$300–1,200	Single‐sample feasible; rapid small‐batch; adaptive sampling	ONT price list; Harvard core; Ali 2024 (7–9 h)
PacBio long‐read (Revio, HiFi)	8–96	1–2 d	8–96	$250–600	$500–1,200	SPRQ chemistry reduces cost per genome; higher yields	UW/Harvard cores; PacBio SPRQ update

**Abbreviations:** BSI, bloodstream infection; Clinical, Clia Laboratory Improvement Amendments; FDA, U.S. Food and Drug Administration; HiFi, high‐fidelity long reads; LDT, laboratory‐developed test; LoD, limit of detection; mNGS, metagenomic NGS; ONT, Oxford Nanopore Technologies; qPCR, quantitative PCR; RUO, research use only; SPRQ, PacBio “Short Protocol for Rare Disease” chemistry; TAT, turnaround time; tNGS, targeted NGS; WGS, whole‐genome sequencing.
Cost range assumes master mix ~$0.30–$0.60/µL, total 5–10 µL per well, and primers/probes ~$0.05–$0.10 per reaction; overhead and labor excluded in “reagent cost,” included in “clinical cost.” Throughput reflects one 384‐well plate; many labs run 2–6 plates/shift with robotics.

* Reagent cost reflects consumables only at moderate scale; does not include labor/overhead/QC/validation.

^†^ Clinical cost varies by setting, interpretation needs, and payer contracts. Short‐read human WGS technical‐only offerings start ~$499, while fully interpreted clinical WGS often bills $3,000–3,400.

^‡^ *Sources:* representative manufacturer information, clinical laboratory fee schedules, academic core rate cards, and peer‐reviewed reports (see “References” column).

#### 2.3.2 Human Whole Genome and Exome Sequencing

The cost of human sequencing has reached new lows in 2023–2025. The cost to sequence a human whole genome (at ~30× coverage) is now *approximately* $600 in many research contexts (Eisenstein 2023; Illumina, Inc. 2023). Major providers have introduced high‐throughput instruments that further slash costs – Illumina's NovaSeq X can produce genomes for about $200 each when fully utilized (Eisenstein 2023; Illumina, Inc. 2023; Palmetto 2021). Emerging technologies are pushing this even further: for example, Ultima Genomics has claimed the potential for a $100 genome with its new platform (Eisenstein 2023). It should be noted that these headline prices reflect sequencing at scale and typically exclude ancillary costs like sample preparation, data storage, and interpretation (Eisenstein 2023). Whole‐exome sequencing (WES), which targets ~1–2% of the genome, is correspondingly cheaper – often on the order of a few hundred dollars per sample for research‐grade sequencing, owing to less sequencing throughput required (Table 1).

Clinically, sequencing is more expensive due to stringent quality and interpretation requirements. A recent analysis estimated the fully loaded cost of a clinical whole‐genome test at about $3,400 per sample (Eisenstein 2023), which includes sequencing reagents, instrument amortization, bioinformatics processing, data storage, and the critical labor of clinical interpretation. Clinical exome sequencing tests are typically offered at lower prices than WGS (roughly half the cost of a genome or less, depending on the provider), reflecting the smaller data burden, though they still often range in the high hundreds to around a thousand dollars per sample in 2025. Overall, while a “$100 genome” is on the horizon for raw sequencing, real‐world clinical genome tests remain in the low‐ to mid‐four‐figure range when all cost components are considered (Table 1).

#### 2.3.3 Metagenomic NGS for Infectious Disease Diagnosis

Untargeted metagenomic sequencing of clinical samples (mNGS) – e.g. sequencing DNA/RNA directly from blood, CSF, or other specimens to identify pathogens – has become an invaluable but relatively costly diagnostic approach. Reported consumables costs for a metagenomic sequencing workflow vary widely (roughly $130 up to $600+ per sample in different research reports) (Govender et al. 2021), due to differences in sequencing depth and sample preparation methods. In practice, current clinical mNGS services are expensive: for example, the UCSF mNGS test for meningitis/encephalitis has an estimated cost of around $3,000 per sample (2024) (Benoit et al. 2024). Other clinical implementations and commercial providers quote prices typically in the $1,000–$2,500 range per test for pathogen‐agnostic NGS diagnostics (often ordered only after extensive routine testing is unrevealing) (Govender et al. 2021; Benoit et al. 2024).

These higher costs reflect the need for deep sequencing (often tens of millions of reads) to detect low‐abundance pathogens, comprehensive analysis pipelines, and interpretation by skilled clinicians or microbiologists. Opportunities to reduce mNGS costs include automation and batching multiple samples per run to leverage economies of scale (Benoit et al. 2024), as well as technical advances like host DNA depletion to decrease the required sequencing depth. Nonetheless, as of 2025, metagenomic NGS remains a relatively resource‐intensive test reserved for complex diagnostic dilemmas, due to its substantial per‐sample cost compared to targeted tests (Table 1).

#### 2.3.4 Multiplexing and Cost Factors

Across all NGS applications, multiplexing (pooled sequencing) is key to reducing the cost *per sample*. Fixed expenditures, such as flow cells and run time, can be shared by sequencing many libraries together. There is an inverse relationship between the number of samples in a run and the per‐sample cost (Waggle et al. 2024). For instance, in one implementation, sequencing 96 bacterial genomes simultaneously on a mid‐throughput instrument brought the reagent cost down to about $48 each, whereas sequencing only a handful of samples would drive up the per‐sample cost to the higher end of the range (Waggle et al. 2024). Human genome sequencing likewise only achieves the advertised $200‐or‐less cost when large batches are processed on ultrahigh‐throughput sequencers at near full capacity (Waggle et al. 2024; Eisenstein 2023). Another factor is library preparation: typical library prep kits and automation add roughly $50–$100 per sample in reagents (at lower scale), though high‐volume methods and kit bulk discounts can drop this cost substantially (e.g., <$60 in reagents per sample for 96‐sample preparation kits (Waggle et al. 2024; Eisenstein 2023; Govender et al. 2021; Benoit et al. 2024; Runheim et al. 2023) (Table 1).

Labor and data analysis are non‐negligible contributors as well, particularly in clinical settings. Skilled personnel must perform DNA extraction, library construction, run monitoring, and downstream bioinformatics; when factored in, personnel can add tens of dollars per sample even in an efficient workflow (Waggle et al. 2024). Furthermore, clinical laboratories incur costs for data storage (genomic data files are large), regulatory compliance, and comprehensive interpretation of results (Runheim et al. 2023). All of these elements mean that the research‐use cost of sequencing (pure reagents) is often substantially lower than the clinical cost per reportable result, which must include labor and infrastructure. The ongoing trend, however, is that improvements in chemistry, throughput, and automation are steadily driving down both reagent and hands‐on costs, continuing to make NGS more accessible and cost‐effective in both research and clinical domains (Waggle et al. 2024; Eisenstein 2023; Govender et al. 2021; Benoit et al. 2024; Runheim et al. 2023) (Table 1).

#### 2.3.5 Cost Burden In Practice

In current U.S. practice, comprehensive clinical metagenomic sequencing can cost on the order of ~$3,000 per sample (e.g., UCSF CSF mNGS), (Benoit et al. 2024), while plasma microbial cell‐free DNA assays are typically priced around $2,000–$2,200 (Sutton et al. 2024; Financial Post 2020). These charges are materially higher than single‐pathogen PCRs and cultures and can be prohibitive outside high‐income settings, even when clinically justified—underscoring the need for explicit coverage pathways to avoid shifting costs to patients or hospitals (Benoit et al. 2024; Financial Post 2020).

### 2.4 Insurance Reimbursement Barriers

Insurance reimbursement policies remain a significant challenge to NGS adoption. Current Procedural Terminology (CPT) codes favor PCR‐based testing, allowing for single‐pathogen tests to be billed under codes such as CPT 87471–87801, which reimburse between $50 and $150 per test (American Medical Association 2025). Large multiplex PCR panels have specific codes, such as CPT 87633, which cover respiratory panels detecting 12–25 pathogens. However, insurance companies often restrict reimbursement for panels testing more than five targets, limiting broader adoption (EmblemHealth 2023). For multiplex PCR panels, payers rarely reimburse each organism separately. CMS guidance instructs laboratories to bill a single high‐level code (e.g., CPT 87633 for 12–25 pathogens), whereas private insurers may down‐code panels > 5 targets to the price of five single‐plex assays (Palmetto 2021). This cap limits the financial incentive to adopt large panels and partly explains why laboratories still run multiple single‐plex PCRs in parallel.

While 384‐well multiplex qPCR LDTs can markedly reduce reagent spend, payer policies often cap reimbursement for higher‐plex assays (e.g., respiratory panels), so realized margins depend on local LCDs and contract terms rather than technical throughput alone.

For NGS‐based tests, reimbursement remains highly inconsistent. Some proprietary laboratory analyses (PLA) codes exist for metagenomic NGS (mNGS) tests, such as the Karius Test (0152U), but these tests are priced at around $2,000 and are frequently denied reimbursement (American Medical Association 2025). Additional PLA codes, such as 0223U and 0225U, have been established for specific NGS applications, but widespread coverage determinations remain limited. A few regional payers are beginning to recognize the clinical utility of metagenomic NGS: for example, California's Medi‐Cal program recently approved limited reimbursement for mNGS in cases of suspected encephalitis or meningitis, following evidence that this approach improves patient outcomes (Wilson et al. 2019), marking an important precedent in U.S. policy.

Although still narrow in scope, such decisions demonstrate that policy shifts toward NGS coverage are possible when supported by clinical efficacy data. This reimbursement landscape creates a paradoxical situation: while NGS offers potential cost savings through comprehensive testing and improved antimicrobial stewardship, the lack of established billing mechanisms prevents its adoption. Healthcare systems are effectively incentivized to perform multiple sequential PCR tests rather than a single, more comprehensive NGS assay (Rossoff et al. 2019).

## 3 Antimicrobial‐Resistance Detection – Clinical Utility and Limitations

Next‐generation sequencing has opened new possibilities for detecting antimicrobial resistance (AMR) determinants in pathogens, but its clinical utility for guiding therapy must be viewed alongside important limitations. Traditional phenotypic susceptibility testing (culture‐based MIC determination) remains the gold‐standard recommended by CLSI and EUCAST for clinical decision‐making (Palmetto 2021). WGS‐based resistance prediction offers several advantages: it can identify a broad array of known resistance genes or mutations in a single analysis, including those for which no routine phenotypic test exists (Rose et al. 2023). It also enables AMR detection in organisms that are slow‐growing or uncultivable, and can uncover novel or unexpected resistance elements (by comparison to databases) that phenotypic methods might miss (Rose et al. 2023). Notably, WGS provides precise information on the presence of specific resistance mechanisms (e.g., beta‐lactamase genes, target site mutations), which can help in understanding and tracking the epidemiology of resistance. In specialized applications such as Mycobacterium tuberculosis, WGS‐based drug resistance prediction has demonstrated high sensitivity and is already used in some reference labs to supplement or even accelerate phenotypic AST, since culture for TB is very slow (Hassall et al. 2024).

NGS delivers two distinct advantages for AMR work‐up: (i) detection of canonical resistance genes that PCR panels overlook, and (ii) discovery of novel or unexpected mechanisms during outbreaks or treatment failure investigations (Ellington 2023; Sherry 2025). For example, *gyrA* codon 83 (S83L, S83Y) and codon 87 (D87N/Y) mutations, together with *parC* S80I in Enterobacterales, raise ciprofloxacin MICs into the CLSI “non‐susceptible” range (>0.5 µg mL⁻¹) and are accurately captured by WGS (Linda 2024). Likewise, *mexR* frameshifts that up‐regulate MexAB‐OprM efflux in *Pseudomonas aeruginosa* often go undetected by cartridge PCR but appear in resistome calls.

Despite these strengths, genotype‐to‐phenotype concordance is imperfect. A 2023 multi‐centre evaluation of 1,256 Enterobacterales isolates showed WGS correctly predicted carbapenem susceptibility in 96% but identified only 22–50% of phenotypically resistant, carbapenemase‐negative strains because porin‐loss and efflux mutations are poorly modeled Rose et al. 2023). EUCAST therefore recommends WGS be used as an adjunct, not a replacement, for phenotypic AST until genomic breakpoints and comprehensive databases are formalized (Rossen 2018).

Unlike phenotypic tests that directly measure growth in response to antibiotics, sequencing can only infer resistance by detecting genes or mutations associated with resistance (Hassall et al. 2024). This distinction leads to important caveats: the presence of a resistance gene does not always equate to phenotypic resistance, and conversely, phenotypic resistance may be present even when no known resistance genes are detected (Hassall et al. 2024). For example, a resistance gene might be present but not expressed or functional (some bacteria carry silent resistance genes that are not phenotypically active under test conditions) (Hassall et al. 2024). On the other hand, an isolate could exhibit resistance via an unknown mechanism or a complex regulatory change that is not captured by current resistance gene databases, yielding a false susceptible prediction by WGS (Hassall et al. 2024; Mmatli et al. 2025a, 2025b).

Studies continue to find cases of genotype–phenotype discordance. For instance, one comparison of WGS‐predicted versus observed drug resistance in Enterobacterales found excellent concordance for several antibiotics (100% genotype prediction sensitivity for ciprofloxacin, gentamicin, etc.), but poor sensitivity (only ~22–50%) for carbapenem resistance because some resistant strains lacked the expected carbapenemase genes (Rose et al. 2023). Such discrepancies often arise from resistance mechanisms such as porin mutations or efflux upregulation that are harder to predict from genome sequence alone (Rose et al. 2023; Mmatli et al. 2025a, 2025b). In general, WGS reliably flags known high‐impact resistance genes (e.g. *mecA* in MRSA, *bla*_KPC_ or *bla*_NDM_ carbapenemases, etc.), achieving very high specificity: the presence of these genes usually correlates with resistance phenotype.

The greater challenge is sensitivity: ensuring that every possible mechanism of resistance can be detected or ruled out by the genomic data (Rose et al. 2023). If a resistant phenotype arises from a novel mutation or gene not in the reference database, WGS will not report it. Thus, a WGS “susceptible” call must be treated with caution if the phenotype suggests otherwise, since it could be a false‐negative prediction (Hassall et al. 2024). This is a key reason why phenotypic AST is still required as a backstop in clinical practice.

Another set of challenges lies in data interpretation and standards. Currently, there is no single universally adopted database covering *all* resistance genes; instead, multiple curation efforts (CARD, ResFinder, NDARO, etc.) exist, and they may differ in content (Hassall et al. 2024). Bioinformatics pipelines also vary; there is a lack of standardization in how labs perform quality control, assembly, and gene calling for resistance, which can lead to inconsistent results between centers (Hassall et al. 2024). Unlike for phenotypic tests, there are no established genomic breakpoints to translate genetic findings into clinical susceptibility categories (Hassall et al. 2024). For example, detecting a particular gene might imply resistance, but the “degree” of resistance (and whether an organism should be reported as intermediate vs. fully resistant) is not straightforward to quantify from sequence alone. Likewise, how to handle combinations of resistance determinants is complex, e.g. an isolate might lack any single high‐level resistance gene but harbor several mutations that together raise the MIC. Interpreting such situations requires expert knowledge and often ancillary phenotypic data (Rose et al. 2023; Mmatli et al. 2025a, 2025b). Additionally, WGS analysis must consider factors such as gene copy number (which can affect expression and phenotype) and plasmids or other mobile elements carrying multiple resistance genes (Rose et al. 2023; Mmatli et al. 2025a, 2025b). Long‐read sequencing can help resolve some of these issues by fully assembling plasmids and repeat regions, but it is not yet standard in clinical (Rose et al. 2023; Mmatli et al. 2025a, 2025b).

Clinical laboratories are starting to merge approaches: WGS or targeted‐NGS for rapid rule‐in of high‐confidence resistance genes (e.g., *mcr‐1, bla*_KPC_, *vanA/B*), followed by rapid MIC panels to confirm activity of narrow‐spectrum options. This hybrid algorithm de‐escalated therapy in 47% and escalated appropriately in 23% of sepsis episodes in one prospective study, shortening length of stay by 3.2 days on average (Stewart 2021).

### 3.1 Clinical Context and Guidelines

Because of these issues, current guidelines do not recommend replacing conventional AST with sequencing for routine infection management. The Clinical Laboratory Standards Institute and EUCAST have acknowledged the potential of WGS for AMR detection but maintain that culture‐based AST remains the reference method for now (Hassall et al. 2024). In 2017, EUCAST outlined hurdles to clinical WGS‐AST implementation, including the need for evidence of prediction accuracy across diverse bacteria, defining how to apply clinical breakpoints to genomic data, standardizing pipelines, and creating comprehensive reference databases (Hassall et al. 2024); challenges that largely still apply in 2025. The FDA has not yet cleared any WGS‐based AST as an IVD for antibiotic susceptibility, and regulatory guidance emphasizes demonstrating equivalence to phenotypic testing. Nonetheless, there is *progress*: researchers and professional bodies are actively working on validation and standardization.

For example, CLSI's AST Subcommittee has been exploring WGS applications for resistance through workshops and pilot studies, and some public health laboratories now routinely use WGS for surveillance of AMR (tracking the spread of resistant clones and genes) (Hassall et al. 2024). In specific niches like tuberculosis or gonorrhea, where rapid genotypic prediction can inform therapy, genomic methods are increasingly employed alongside phenotypic tests. Yet even in these cases, results must be interpreted with clinical correlation. It is generally recommended that genotypic AMR predictions be used to supplement, not replace, phenotypic susceptibility testing at this stage (Hassall et al. 2024).

In summary, NGS offers tremendous clinical utility for AMR detection in terms of comprehensive surveillance and the ability to identify known resistance markers in a single assay, potentially faster than conventional methods. It is particularly useful for organisms or sample types where culture is difficult or slow, and for investigating outbreaks or the transmission of resistance genes (genomic data can link isolates and highlight shared resistance elements). It also holds promise for guiding therapy in the future; for instance, by rapidly ruling in key resistance genes in a serious infection, allowing earlier optimization of antibiotics. However, important limitations temper its standalone use: (1) Phenotypic correlation is imperfect as WGS may miss unexpected resistance or overpredict clinically irrelevant ones. Hence, results need confirmation or at least careful validation against phenotypic AST (Rose et al. 2023; Mmatli et al. 2025a, 2025b). (2) Interpretation complexity, i.e., translating raw genomic data into an accurate clinical prediction requires robust databases and expertise, and no consensus framework is yet fully established (Hassall et al. 2024). (3) Regulatory and workflow issues are also major bottlenecks as implementing WGS in a clinical laboratory requires infrastructure for rapid sequencing and analysis, as well as personnel training to analyze results in real‐time, which can be a hindrance compared to the simplicity of disk diffusion or automated MIC panels.

These challenges mean that, as of 2025, WGS is typically used as an adjunct tool for AMR, providing insights into resistance genes and mechanisms and aiding outbreak investigations and surveillance, rather than replacing conventional AST in guiding everyday patient care (Hassall et al. 2024). Going forward, improvements in databases, consensus on interpretation guidelines, and further clinical validation studies (showing where genome‐based predictions can reliably inform therapy) will be crucial. With these advancements, NGS is expected to play an increasingly complementary role, and eventually perhaps a transformative role, in how we detect and interpret antimicrobial resistance, but for now it must be paired with phenotypic methods to ensure comprehensive and safe patient care.

### 3.2 Syndromic Testing and Polymicrobial Infections

NGS provides significant advantages in diagnosing polymicrobial infections, where multiple pathogens contribute to disease pathology. Traditional PCR requires multiple independent assays, increasing costs and sample processing time. NGS, particularly targeted sequencing, enables simultaneous detection of bacteria, viruses, fungi, and parasites in a single assay, making it an ideal solution for syndromic testing (Altindiş and Kahraman Kilbaş 2023; Relich 2025).

For gastrointestinal (GI) infections, tNGS can identify multiple bacterial (e.g., *Clostridioides difficile*, *Campylobacter* spp.), viral (e.g., norovirus, rotavirus), and parasitic pathogens (e.g., *Cryptosporidium* spp.) simultaneously, reducing diagnostic uncertainty (Angel et al. 2025; Fernandez‐Cassi et al. 2020). In respiratory infections, where co‐infections are common, NGS offers broader pathogen coverage compared to PCR, capable of distinguishing between viral pathogens and bacterial superinfections that require different treatment approaches (Langelier et al. 2018).

Similarly, in skin and soft tissue infections (SSTI), NGS improves the detection of polymicrobial infections, providing essential data for antimicrobial stewardship (J, D. B. and E, M). Its application in sexually transmitted infections (STIs), bacterial vaginosis (BV), aerobic vaginitis (AV), and urinary tract infections (UTIs) further demonstrates its capability in capturing co‐infections more comprehensively than PCR (Cartwright 2013).

Several studies have demonstrated NGS's superior performance in detecting polymicrobial infections. In a study of 44 clinical samples, tNGS identified all pathogens detected by culture and PCR, plus an additional 35% of clinically relevant organisms missed by conventional methods (Quainoo et al. 2017; Bearzatto et al. 2025; Hayden et al. 2025). This enhanced detection capability is particularly valuable in immunocompromised patients, where rare or opportunistic pathogens may be present but missed by targeted PCR panels (Wilson et al. 2019).

## 4 Overcoming Challenges in NGS Implementation

### 4.1 Reducing Cost and Turnaround Time

Recent technological advances have addressed some of the primary barriers to NGS adoption. Newer platforms, such as the Illumina iSeq. 100 (and MiSeq) and the Thermo Fisher Genexus, offer reduced sequencing times and simpler workflows, bringing TAT closer to that of PCR (Quainoo et al. 2017; Bearzatto et al. 2025; Hayden et al. 2025). The development of optimized library preparation protocols has further streamlined the process, with some workflows now requiring less than 24 h from sample to result (Charalampous et al. 2019).

A key avenue for lowering per‐sample expense is multiplexing—running multiple samples simultaneously on a single flow cell or chip. By carefully designing or synthesizing custom indexing primers (barcodes), laboratories can pool dozens of patient specimens together, drastically bringing down the reagent cost per sample. Commercial index kits can cost $10–$20 per sample, whereas custom‐synthesized indices can be $1 or less per sample if done in bulk. The trade‐off is that a more “homemade” approach requires optimization and validation but can be very cost‐effective at scale. This approach is particularly effective for targeted sequencing, where focused amplification of relevant genomic regions allows for higher sample throughput without sacrificing depth of coverage (Hardwick et al. 2017).

Cloud‐based bioinformatics solutions have also emerged to address the computational challenges of NGS data analysis. These platforms offer user‐friendly interfaces and automated pipelines that reduce the need for specialized bioinformatics expertise, making NGS more accessible to clinical laboratories (Grubaugh et al. 2019).

### 4.2 Influence of Amplicon Size and Coverage Depth

Another critical variable influencing cost and performance is amplicon size (75–1000 bp). Smaller amplicons (75–200 bp) can be amplified and sequenced more cheaply and quickly, but may not capture enough sequence variation to definitively distinguish between closely related strains. In contrast, longer amplicons (500–1000 bp) provide more genetic context but often require specialized platforms or additional assembly steps, increasing both cost and turnaround time. Likewise, coverage depth (e.g., 30× vs. 100×) determines how reliably low‐level infections can be detected; higher coverage bolsters sensitivity and variant detection but directly drives up sequencing costs.

## 5 Addressing Sensitivity Limitations

While NGS offers broad coverage, PCR often demonstrates superior sensitivity for detecting low‐abundance pathogens. This limitation can be addressed through improved sample preparation techniques, such as host DNA depletion and pathogen enrichment (Marotz et al. 2018). Additionally, targeted sequencing approaches that focus on clinically relevant genomic regions can achieve sensitivity comparable to PCR while maintaining broader coverage (Thoendel et al. 2016). A hybrid diagnostic approach may be optimal in many clinical scenarios: using NGS for comprehensive initial screening, followed by PCR for confirmation or quantification of specific pathogens. This strategy leverages the strengths of both technologies while mitigating their limitations (Miller et al. 2019). For instance, a commercial hybrid‐capture respiratory mNGS assay achieved an LoD of 20 viral genome copies mL⁻¹ for SARS‐CoV‐2, matching RT‐qPCR sensitivity after host‐DNA depletion and 10 million reads per sample (Eisenstein 2023; Illumina Pitches $200 Genomes With New Line of DNA Sequencers n.d.; Illumina's Revolutionary NovaSeq 2023).

### 5.1 Platform‐Specific Differences

When choosing among leading platforms such as Illumina (MiSeq, iSeq), Thermo Fisher Ion Torrent (S5, Genexus), or emerging 4th generation technologies (PacBio, Oxford Nanopore), laboratories must balance cost per gigabase, run times, and read lengths. Illumina's systems often excel in data quality and throughput, making them ideal for large panels, whereas Ion Torrent equipment may be favored when laboratories want faster runs and simpler, turnkey workflows. Nanopore and PacBio devices can deliver long reads or near‐real‐time sequencing, but they remain costlier or less clinically validated in some regions. Each platform's capacity, error profile, and kit ecosystem significantly influence cost‐effectiveness and turnaround time for targeted NGS. Only a few of the listed systems currently hold FDA 510(k) clearance for infectious‐disease NGS (e.g., Thermo Fisher's Ion Torrent S5 using the CE‐IVD accredited “Oncomine Pan‐Pathogen Research Assay”). Most clinical labs therefor,e validate assays as CLIA‐high‐complexity LDTs, following NY‐DoH and CAP guidelines that require ≥ 95% agreement with reference methods across ≥ 50 positive and ≥ 50 negative samples per target (Eisenstein 2023; Illumina Pitches $200 Genomes With New Line of DNA Sequencers n.d.; Illumina's Revolutionary NovaSeq 2023; BioSpace 2024).

### 5.2 Fourth‐Generation, Long‐Read Options for Low‐Volume Labs

A practical advantage of Oxford Nanopore is the ability to run a single urgent sample without waiting to batch, delivering same‐day ID/AMR insights when paired with rapid kits; studies repeatedly show 7–12 h end‐to‐answer in sepsis/BSI pipelines. Adaptive sampling can deplete human DNA or enrich microbial targets on‐the‐fly to boost sensitivity and speed. PacBio Revio yields highly accurate HiFi reads and supports pooled microbial WGS at ~$400–$600 per isolate at academic cores, with long‐read human WGS approaching <$500 per genome at scale under the SPRQ workflow. Together, these long‐read modalities complement short‐read tNGS for contexts where rapid single‐sample turnaround or genome closure/plasmid context matters; however, both vendors' instruments are labeled RUO (not IVD), so results used clinically generally fall under LDTs in CLIA labs (BioSpace 2024; Cheng et al. 2022; Ali et al. 2024; Moragues‐Solanas et al. 2024).

## 6 Regulatory and Validation Pathways

For widespread adoption, NGS‐based infectious disease diagnostics must navigate complex regulatory pathways. The FDA has issued guidance for NGS‐based in vitro diagnostics, but many tests are currently developed as laboratory‐developed tests (LDTs) under CLIA regulations (US Food and Drug Administration n.d.). Standardized validation protocols that address sensitivity, specificity, reproducibility, and limit of detection are essential for regulatory compliance and clinical confidence (College of American Pathologists 2025).

Implementing an NGS‐based infectious disease assay requires rigorous validation covering analytical sensitivity, specificity, limit of detection, reproducibility, and comprehensive bioinformatics pipeline checks. Guidelines from the New York Department of Health, the College of American Pathologists (CAP), and the Clinical Laboratory Improvement Amendments (CLIA) typically demand showing consistent detection of multiple pathogens, accurate identification of drug‐resistance genes, and ongoing quality controls. Unlike single‐target PCR, a broad multiplex NGS panel may have dozens (or hundreds) of amplicons to validate, making the process more involved—and thus more time and cost‐intensive—but crucial for ensuring robust clinical adoption.

Bioinformatics pipeline validation presents a particular challenge, as updates to reference databases and algorithm improvements require ongoing assessment. Establishing quality metrics and control procedures for NGS data analysis is critical for ensuring consistent and reliable results (Roy et al. 2018).

Engagement with regulatory agencies, professional societies, and standards organizations is necessary to develop consensus guidelines for NGS validation in infectious disease diagnostics. These guidelines should balance rigor with practicality to enable innovation while ensuring patient safety (Gargis et al. 2015).

## 7 Global Perspective: International NGS Implementation

While insurance reimbursement barriers have slowed NGS adoption in the United States, several countries have successfully integrated NGS into routine infectious disease diagnostics through various healthcare funding models and strategic implementation approaches.

### 7.1 European Models of Implementation

The United Kingdom's National Health Service (NHS) has pioneered systematic NGS implementation through its Genomic Medicine Service, which includes pathogen genomics for clinical and public health applications (Coll et al. 2017). The NHS 100,000 Genomes Project established infrastructure and expertise that has extended to infectious disease diagnostics, with centralized sequencing hubs serving regional networks. This nationwide approach allowed for economies of scale and standardized workflows that significantly reduced per‐test costs (Peacock et al. 2018).

Denmark has implemented a national genomic surveillance network where WGS is routinely used for foodborne outbreak investigations, antimicrobial resistance surveillance, and hospital infection control (Petersen et al. 2015). Their model leverages centralized bioinformatics resources while distributing sequencing capabilities across regional centers, creating a sustainable funding structure through their universal healthcare system.

In Germany, the Robert Koch Institute coordinates a national network for genomic surveillance where NGS is routinely deployed for infectious disease diagnosis and outbreak investigation. Their model includes reimbursement for NGS through specific billing codes in their statutory health insurance system, particularly for complex cases where conventional diagnostics have failed or for specific high‐priority pathogens (Kohl et al. 2014).

### 7.2 Asia‐Pacific Implementation Strategies

Singapore has integrated NGS into its public health infrastructure through the National Public Health Laboratory, which routinely employs genomic approaches for outbreak investigation and surveillance (Ko et al. 2022). Their model benefits from centralized funding and coordination, allowing for rapid deployment of NGS technologies during outbreaks without reimbursement barriers.

Australia has developed a national framework for pathogen genomics that includes standardized approaches to NGS implementation and reimbursement through its Medicare Benefits Schedule (Sherry et al. 2019). Their model focuses on specific clinical scenarios where NGS demonstrates clear cost‐effectiveness compared to conventional testing, allowing for targeted implementation that addresses priority health needs.

Japan has incorporated NGS testing for infectious diseases into their universal health insurance system through a staged implementation approach, beginning with specific pathogens and clinical scenarios before expanding coverage (Tohya et al. 2022). Their methodical approach to evaluation and reimbursement provides a model for evidence‐based expansion of NGS coverage.

In contrast to the largely private, multi‐payer framework in the United States, many high‐income countries (e.g. Canada, Australia, New Zealand, and much of Europe) operate publicly funded or universal healthcare systems, simplifying adoption of advanced diagnostics like NGS by enabling national‐level reimbursement policies and facilitating broad population access (Henríquez 2025; Valencia‐Shelton 2024).

### 7.3 Public‐Private Partnerships

Several countries have leveraged public‐private partnerships to accelerate NGS adoption. South Korea's collaboration between the Korea Disease Control and Prevention Agency and commercial laboratories has enabled routine use of NGS for antimicrobial resistance surveillance and outbreak investigation (Kim et al. 2022). This model distributes costs between public health objectives and clinical applications, creating a sustainable funding structure.

In Brazil, a network of public and private laboratories coordinated by the Ministry of Health employs NGS for surveillance of emerging infectious diseases, with costs covered through a combination of public health funding and fee‐for‐service reimbursement for clinical applications (Maljkovic Berry et al. 2020). This hybrid approach has expanded access to NGS testing while building capacity for future implementation.

### 7.4 Lessons for Reimbursement Reform

International implementation models offer valuable lessons for addressing reimbursement barriers:

1. **Prioritized Implementation:** Many countries have successfully started with specific high‐impact applications (e.g., AMR detection, complex cases) where NGS demonstrates clear advantages over conventional testing (Henríquez 2025; Valencia‐Shelton 2024).
2. **Centralized Infrastructure:** National or regional sequencing centers that serve multiple healthcare facilities can achieve economies of scale while maintaining standard quality metrics (Tran et al. 2023; Buchanan et al. 2025).
3. **Tiered Reimbursement Models:** Some countries have implemented tiered payment systems where reimbursement rates vary based on the complexity and comprehensiveness of the NGS test, reflecting the true value of the diagnostic information provided.
4. **Evidence‐Based Expansion:** Systematic collection of outcomes data to demonstrate clinical utility and cost‐effectiveness has supported the gradual expansion of NGS reimbursement in several healthcare systems (Trivett et al. 2025).

These international examples demonstrate that with appropriate reimbursement mechanisms and implementation strategies, NGS can be successfully integrated into routine infectious disease diagnostics, providing valuable lessons for policy reform in the United States.

## 8 The Path Forward: Policy Recommendations

### 8.1 Insurance Policy Reform

To overcome reimbursement barriers, the policy changes below are proposed:

1. Establish dedicated CPT codes for NGS‐based infectious disease testing that reflect the comprehensive nature of these assays and their potential to replace multiple PCR tests (Centers for Medicare and Medicaid Services 2025).
2. Implement value‐based reimbursement models that consider downstream cost savings from improved diagnostic accuracy, reduced antibiotic use, and shorter hospital stays (Porter and Teisberg 2007).
3. Develop coverage criteria based on clinical utility rather than historical precedent, recognizing the evolving nature of diagnostic technologies (Garber and McClellan 2007).

Notably, the American Medical Association's CPT Editorial Panel has begun reviewing new or revised Tier III codes for comprehensive infectious disease NGS tests, indicating slow but ongoing progress toward standardized billing. The AMA CPT Editorial Panel's May 2025 action did not add a rapid whole‐genome sequencing GSP code (the proposal was rejected) and withdrew several genome‐scale items (e.g., oncology WGS, optical genome mapping). New microbiology panel codes were accepted for joint‐infection multiplex testing and an amplified probe STI panel (effective Jan 2026). Thus, no new CPT code currently targets infectious‐disease NGS/mNGS broadly; most ID sequencing continues to rely on LDT frameworks, PLA codes, or panel‐specific CPTs (American Medical Association 2025). While rapid WGS was not adopted, two new microbiology panel codes effective Jan 2026 offer immediate billing pathways for joint infection and STI panels; laboratories should align LDT validations and payer dossiers for these panels while re‐proposing rapid WGS with stronger utilization and outcomes evidence.

Although these efforts remain fragmented, they underscore the need for cohesive national guidance that fully recognizes the broad scope of NGS‐based pathogen detection.

### 8.2 Bioinformatics and Billing Considerations

Beyond generating results, bioinformatic deconvolution is critical for billing and reimbursement strategies. A single NGS run may test for dozens of pathogens simultaneously, but laboratories often need to submit separate billing codes for each clinically relevant pathogen identified. Robust bioinformatics pipelines can segregate each organism detected into individual results that align with CPT codes for single‐pathogen PCR tests or smaller panel codes, potentially allowing multiple reimbursements from a single multiplex run. This approach can help labs secure coverage for the overall cost of NGS.

Automated bioinformatics pipelines can generate discrete line‐item reports for each pathogen, effectively mapping NGS read counts to existing single‐target or panel codes (Roy et al. 2018; Gargis et al. 2015). This level of granularity enables laboratories to “unbundle” a comprehensive NGS run into multiple reimbursable results, provided each target meets medical necessity criteria. As the Association for Molecular Pathology (AMP) and the College of American Pathologists (CAP) refine NGS reporting guidelines, these approaches will become increasingly standardized and accepted (Gargis et al. 2015).

#### 8.2.1 Clinical Implementation Strategies

Healthcare systems can facilitate NGS adoption by:

- Adopt a reflex algorithm: start with syndromic PCR; reflex to targeted‐NGS when PCR negative or polymicrobial infection suspected (Lewandowski et al. 2019).
- Batch specimens daily and apply 24‐h library‐prep “midnight protocol” to keep turnaround < 48 h (Oude Munnink et al. 2020).
- Pool sequencing across hospital networks via a regional NGS hub to gain economies of scale (Oude Munnink et al. 2020).
- Embed real‐time decision‐support software that converts resistome data into WHO‐AWaRe antibiotic categories for stewardship review (Shortliffe and Sepúlveda 2018).

#### 8.2.2 Research Priorities

To strengthen the evidence base for NGS in infectious disease diagnostics, research should focus on

- Quantify cost‐utility of tNGS versus multiplex PCR in prospective RCTs across sepsis, meningitis, and prosthetic‐joint infections (Ivy et al. 2018).
- Define genomic breakpoints and confidence thresholds for WGS‐AST in at least five priority pathogens by correlating ≥ 5000 matched genotype–phenotype pairs (Eccles and Mittman 2006).
- Develop open‐source, regulator‐ready pipelines that auto‐update AMR databases while maintaining locked versions for accreditation (Goldberg et al. 2015).
- Model payer budget‐impact of CPT‐coded NGS bundles compared with current multi‐plex PCR reimbursement caps.

## 9 Conclusion

NGS, particularly targeted amplicon sequencing, offers unparalleled advantages in infectious disease diagnostics by enabling broad pathogen detection, antimicrobial resistance profiling, and syndromic testing in a single assay. However, insurance reimbursement remains a major barrier preventing widespread adoption. To ensure cost‐effectiveness, multiplexing strategies, emerging NGS platforms, and workflow optimization must be leveraged.

The insurance industry must modernize reimbursement policies to recognize NGS's superior clinical utility, ultimately reducing healthcare costs and improving patient outcomes. Future research should focus on demonstrating real‐world cost savings and advocating for policy changes that align with the evolving landscape of infectious disease diagnostics.

Adopting and providing CPT codes for tNGS diagnostics in infectious diseases will ultimately benefit all stakeholders, insurers, diagnostic companies, clinicians, and patients, as more samples will be multiplexed, driving down the cost of diagnosis and providing a higher resolution and breadth of infectious disease detection. The time has come to align reimbursement policies with technological innovation, ensuring that patients have access to the most comprehensive and effective diagnostic tools available. As demonstrated by successful international implementation models, with appropriate funding structures and strategic deployment, NGS can transform infectious disease diagnostics at scale, improving both individual patient care and public health outcomes. As barriers fall, tNGS and metagenomic sequencing are poised to transition from “last‐resort send‐out” to first‐line diagnostics.

## Author Contributions

John Osei Sekyere, B. Pharm, M. Phil, Ph.D.: conceptualization, drafting, writing, reviewing, supervision, funding, software/figure generation, editing, and formatting.

## Ethics Statement

The author has nothing to report.

## Conflicts of Interest

The author declares no conflicts of interest.

## Transparency Statement

The author is an NGS Scientist at Medical Diagnostic Laboratories LLC, a diagnostic laboratory serving clinicians across the United States. The decision to write or publish this study was not influenced by the employers of the author.
